# Research Participation and Hypertension: Examining the Effects of Blood Pressure Monitoring in a Validation Study

**DOI:** 10.1093/geroni/igaf122.2893

**Published:** 2025-12-31

**Authors:** Mitchell Roberts, Edward Hammel, Erica Sappington, Roma Tremblay, Alyssa Persad, Samantha Hoffman, Cyriah Carey, Carla VandeWeerd

**Affiliations:** University of Florida, Gainesville, Florida, United States; UF Health - Precision Health Research Center, The Villages, Florida, United States; UF Health - Precision Health Research Center, The Villages, Florida, United States; UF Health - Precision Health Research Center, The Villages, Florida, United States; UF Health - Precision Health Research Center, The Villages, Florida, United States; UF Health - Precision Health Research Center, The Villages, Florida, United States; UF Health - Precision Health Research Center, The Villages, Florida, United States; UF Health - Precision Health Research Center, The Villages, Florida, United States

## Abstract

Unmanaged BP and Cardiovascular disease (CVD) are leading health concerns among adults 55+ resulting in increased costs and mortality. This research explored whether participation in an observational BP study influenced CVD management. Participation included validation measurements for The Heart Seat (THS), a novel BP device, alongside a gold-standard BP cuff. Participants received their gold-standard BP readings, potentially increasing awareness of their BP status.491 older adults (M:71.9, SD:6.8, range:50–89 years, 50% female) participated in the validation study at UFHealth PHRC- The Villages, FL (UFIRB202400472) from 5/27/24–10/17/24. All participants completed two study visits, providing demographic, BP, and medical history data. Those with systolic BP < 100mmHg (hypotensive) or ≥ 150mmHg (hypertensive) readings were invited for a third visit (V3) to enhance validation data on high/low BP cases. Of the 491 participants, 45(9.2%) reported CVD-related medical history change between 1st and 2nd visits. Changes included following up to receive new hypertension and/or arrhythmia diagnosis, and/or start of novel medications or medication adjustments. Additionally, among 41 participants with high/low BP invited for V3, 16(39%) reported changes post-visit 2, with 13(81%) initiating BP medications. Though research participation can influence health behaviors, this is often overlooked in the design and follow-up of non-interventional studies. 10% of study participants self-reported changes in CVD management following study participation, with higher rates amongst those at BP extremes. Findings highlight the need to better understand how study participation can positively impact long-term health decisions and clinical care even when non-interventional. Implications for policy and practice will be discussed.

